# Technological advances in ocular trabecular meshwork in vitro models for glaucoma research

**DOI:** 10.1002/bit.28182

**Published:** 2022-07-25

**Authors:** Maria Bikuna‐Izagirre, Javier Aldazabal, Leire Extramiana, Javier Moreno‐Montañés, Elena Carnero, Jacobo Paredes

**Affiliations:** ^1^ Tecnun School of Engineering University of Navarra San Sebastián Spain; ^2^ Biomedical Engineering Center University of Navarra Pamplona Spain; ^3^ Departamento de oftalmología Clínica Clínica Universidad de Navarra Pamplona España

**Keywords:** aqueous humor outflow, glaucoma, scaffolds, tissue‐engineering, trabecular meshwork

## Abstract

Glaucoma is the leading cause of irreversible blindness worldwide and is characterized by the progressive degeneration of the optic nerve. Intraocular pressure (IOP), which is considered to be the main risk factor for glaucoma development, builds up in response to the resistance (resistance to what?) provided by the trabecular meshwork (TM) to aqueous humor (AH) outflow. Although the TM and its relationship to AH outflow have remained at the forefront of scientific interest, researchers remain uncertain regarding which mechanisms drive the deterioration of the TM. Current tissue‐engineering fabrication techniques have come up with promising approaches to successfully recreate the TM. Nonetheless, more accurate models are needed to understand the factors that make glaucoma arise. In this review, we provide a chronological evaluation of the technological milestones that have taken place in the field of glaucoma research, and we conduct a comprehensive comparison of available TM fabrication technologies. Additionally, we also discuss AH perfusion platforms, since they are essential for the validation of these scaffolds, as well as pressure–outflow relationship studies and the discovery of new IOP‐reduction therapies.

## Graphical abstract

A comprehensive comparison between existing tissue‐engineering fabrication technologies for trabecular meshwork (TM) mimicking is studied in this review. Moreover, aqueous humor perfusion platforms are evaluated due to their fundamental value to validate these scaffolds and perform outflow studies. Appropriate mimicking of the native TM will not only provide repeatability to many studies but will also allow the understanding of the biological and physiological issues related to glaucoma that are, as of yet, unanswered.

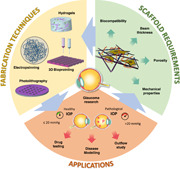

## 1 INTRODUCTION

Glaucoma is the leading cause of irreversible blindness worldwide. It afflicts well over 70 million people and it is estimated that this number will rise to 111.8 million in 2040 (Tham et al., 2014). Given that the first stage of the illness is asymptomatic, the number of affected individuals may actually be substantially larger than the number of confirmed cases, which further cements the impact this illness is having on populations across the globe (Traverso et al., 2005).

Primary open‐angle glaucoma (POAG) represents 90% of glaucoma cases worldwide and is most common among black people, followed by Hispanic individuals (Tham et al., 2014; Weinreb et al., 2016). It is characterized by the functional deterioration of the optic nerve, including the optic nerve head and the retinal ganglion cells. Hereditary conditions (first‐degree relatives) and high intraocular pressure (IOP) are determinant factors for the progression of this sickness. Additionally, age (Gong et al., 1992) is also a significant risk factor for glaucoma development, together with high blood pressure levels, high myopia, and prolonged treatment with ocular steroids. Also, the male sex has been shown to have a 30% increased risk over the female sex (Weinreb et al., 2016).

**Figure 1:**
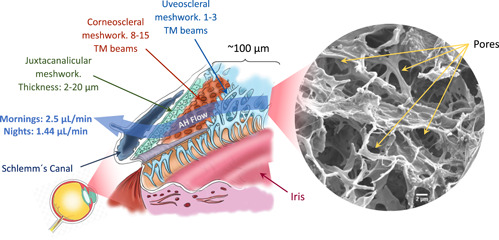
Location of the trabecular meshwork (TM). The anatomical structure of this tissue formed by three layers: uveal, corneoscleral, and juxtacanalicular meshwork. Scanning electron microscopy (SEM) image of a real decellularized TM tissue (author's own work). Scale bar: 2μm.

Among these factors, the IOP is the most important risk factor. IOP levels are considered normal between 10 and 20 mmHg, with the prevalence of POAG increasing dramatically when these levels exceed 20 mmHg (Tamm & Fuchshofer, 2007; Weinreb et al., 2016). The trabecular meshwork (TM) outflow pathway (Figure 1) provides resistance to aqueous humor (AH) outflow. The structures that form this TM pathway, like the inner wall of Schlemm's canal (SC), SC itself, the collecting channels, the aqueous veins, and the TM (Tamm, 2009) build up the IOP. The TM is estimated to be responsible for 90% of the drainage of the AH from the ocular cavity (Goel et al., 2010; Grant, 1958). Changes in its physical properties, such as increase in stiffness (from 4 to 80 kPa; Last et al., 2011; Liu et al., 2018; Wang et al., 2017), alterations in extracellular matrix (ECM; Keller et al., 2009) protein expression or loss in its reparative capacity, have been shown to be associated to AH outflow difficulties. These changes in physical properties provoke an increase in IOP, which ends up deteriorating the optic nerve and retinal ganglion cells (Carreon et al., 2017). In addition, circadian rhythms modulate flow rates across the membrane in response to daily variations of the IOP (Goel et al., 2010; Tamm, 2009). Figure 1 shows a representation of the TM, its layered structure, the drainage flow direction as well as a scanning electron microscopy (SEM) image of a human TM from a healthy donor. This tiny triangle‐form porous tissue is approximately 100 μm thick and 694.9 ± 109.0 and 713.2 ± 109.6 μm in length for both females and males, respectively (Abu‐hassan et al., 2014; Kasuga et al., 2013; Yan et al., 2016). It can be understood as the amalgamation of three different regions, whose characteristics are summarized in Table 1 (Acott & Kelley, 2008; Gong et al., 1992; Grant, 1958; Keller et al., 2009; Tamm, 2009).

**Table 1:** Characteristics and structure of the human TM

Layer	Location	Thickness (μm)	Morphology	ECM composition	Cell type	Authors
Uveoscleral meshwork	Extends from the iris root and ciliary body to the Schwalbe's line	25–27	3–4 beam layers. Open spaces between beams Highly porous	Collagen I, III and elastic fibers	Endothelium macrophage	Tamm (2009), Tamm and Fuchshofer (2007),Buffault et al. (2020)
Corneoscleral meshwork	From the anterior wall of the scleral sulcus to the scleral spur	2–15	8–15 layers. Open spaces between beams Highly fenestrated structure and porosity	Collagen I, III and elastic fibers	Endothelium macrophage	Tamm (2009),Grant (1958), Tamm and Fuchshofer (2007),Buffault et al. (2020)
Juxtacanalicular meshwork	Its outermost portion corresponds to the inner wall of SC	2–20	2–5 layers of scattered cells Compact and amorphous	Covered with an endothelial layer, elastic fibers of collagen IV, laminin, fibronectin, hyaluronic acid	Fibroblast smooth muscle cell	Grant (1958), Tamm (2009),Gong et al. (1992),Buffault et al. (2020)

Abbreviations: ECM, extracellular matrix; SC, Schlemm's canal; TM, trabecular meshwork.

In the past, different animal models have been employed to unveil the relationship between the TM and the AH outflow (in vivo models such as monkeys, dogs, cats, rodents, pigs, and several other species; Bouhenni et al., 2012; Mao et al., 2011; Peche & Eule, 2018; Rasmussen & Kaufman, 2005). These models provide an entire ocular globe to analyze different hypotheses and to test a number of different drugs. However, animal models have the drawback of exhibiting the well‐known “washout effect.” This effect describes how the eye accommodates to the new physiological conditions with an increase in outflow over time attributed to ECM components removal in outflow pathway tissues (Lei et al., 2011). Furthermore, the unpredictability of glaucoma induction in animal eyes, the need for sophisticated equipment, and the necessity of trained personnel (Bouhenni et al., 2012) make it difficult to conduct glaucoma studies in animals.

Arguably, the most fundamental findings concerning the TM and AH outflow relationship have come from studies based on whole enucleated post‐mortem human eyes. The seminal studies of Morton Grant (Ellingsen & Grant, 1971a; Grant, 1963) are a prime example of such research. Most of Dr. Grants works confirmed that an internal trabeculectomy in enucleated human eyes approximately eliminates 75% of AH resistance (Ellingsen & Grant, 1971b; Johnstone & Grant, 1973; Van Buskirk & Grant, 1973). This result expanded the belief that the principal site of AH resistance lies proximal to the outer wall of SC, near the juxtacanalicular tissue. Dr. Grant also studied the relationship between pressure and outflow resistance, concluding that in human and primate eyes with an intact outflow system, elevated perfusion pressure caused an increase in outflow resistance (Ellingsen & Grant, 1971a; Rosenquist et al., 1989). Ex vivo models have a number of important advantages over other models, such as their preservation of pathway architecture and how they enable analysis in quasi‐perfect physiologic states. However, they are limited in number and their use in the study of steroid‐induced IOP elevation has been infrequent (Clark et al., 1995; Johnson et al., 1990; Rybkin et al., 2017). For these reasons, and despite the amount of time that has been devoted to research this unique tissue and its relation with AH outflow, the scientific community is still unsure regarding which physical and molecular mechanisms drive the malfunction of the TM tissue.

In the last decade, interest in the performance and behavior of TM has increased and a need to engineer novel TM in vitro models has arisen. A number of different approaches have been developed in recent years to successfully create scaffolds for 3D culture that can emulate the TM: photolithography, electrospinning, hydrogel molding, or 3D bioprinting. Appropriate mimicking of the native TM will not only provide repeatability to many studies but it will also allow us to understand the biological and physiological issues related to glaucoma that are, as of yet, unanswered. Moreover, the recreation of AH outflow physiology using artificial TM models as part of engineered perfusion systems may potentially reduce the use of animal and human organ models and boost research in the field toward improving the current understanding of AH outflow mechanisms and developing new drug therapies for glaucoma disease.

In this study, we provide a chronological review of the technological milestones that have furthered the scientific community's understanding of glaucoma disease. Given that the TM plays a pivotal role in the evolution of this malady; it drainages the AH and regulates the IOP (Braunger et al., 2015; Goel et al., 2010), its characteristics should be recreated as closely as possible to the native TM. Thus, current TE fabrication technologies, as well as their respective benefits and drawbacks, are also studied in this review. We also discuss AH perfusion platforms, as they are essential for the validation of these engineered scaffolds and pressure–outflow relationship studies. As a whole, the purpose of this paper is to provide a comprehensive comparison between existing TM scaffolding fabrication methods and those bioreactor systems that have the potential to improve our understanding of AH outflow—pressure relationship and to the mechanisms behind TM malfunctioning.

## 2 TECHNOLOGICAL MILESTONES FOR GLAUCOMA FUNDAMENTS

**Figure 2:**
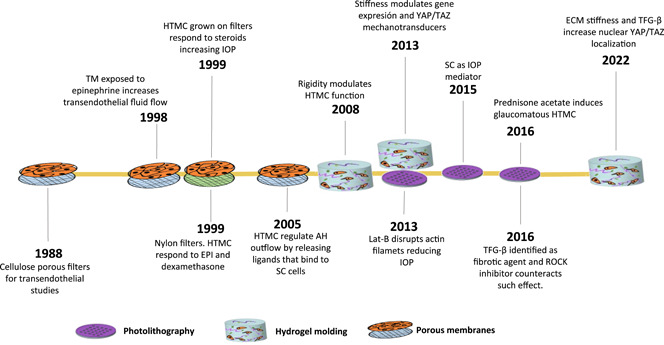
Technology‐based most important milestones regarding glaucoma research

Figure 2 provides a graphical representation of the most important milestones in glaucoma research. In all these works, engineered scaffolds were used to mimic the TM. The first isolation of human trabecular meshwork cells (HTMCs) occurred in 1979 using recent post‐mortem specimens, which ultimately led to a new era in glaucoma research (Polansky et al., 1979). These first studies were based on standard plate cell cultures, which enabled the discovery of HTMC characteristics and identified the components of the ECM (Alvarado et al., 1982; Hernandez et al., 1987; Schachtschabel & Binninger, 1990; Wolffe & Tata, 1984).

Later, in 1988, studies on AH outflow became possible thanks to the use of filters of different materials as TM scaffolds. This study served to study the behavior of HTMC and to see how the cell monolayers can regulate the hydraulic conductivity of the tissue, in other words, how easily the flow can pass through the membrane. Furthermore, these experimental setups served to test the effect of different drugs on the cellular performance. For instance, cellulose ester porous filters were used to quantify the hydraulic conductivity of the tissue once exposed to cytochalasin, an actin filament's polymerization blocker, which causes rapid outflow capacity (Perkins et al., 1988). With the same objective, HTMC were also cultured over nylon filters, which were subjected to different TM‐targeted drugs (EPI and dexamethasone; Dai & Li, 1999). As discovered by Polansky et al. (1981), HTMC grown on the nylon filters responded to steroid treatment, decreasing flow through cell monolayer (referred to in their paper as the transendothelial conductivity) and causing IOP elevation (Underwoord et al., 1999). These filters also revealed that when HTMC is exposed to epinephrine, transendothelial fluid flow increases lowering the IOP (Alvarado et al., 1998). During the first decade of the XXIst century, these membranes were used to verify the hypothesis that TM endothelial cells regulate AH outflow by actively releasing ligands that upon binding to SC, increase transendothelial flow, and thereby facilitate the egress of the liquid (Alvarado et al., 2005; Ryan et al., 2010).

Simultaneously, from the year 2000 onwards, novel tissue engineering (TE) fabrication methods start replacing these filter membranes. Despite the fact that these new manufacturing techniques present their own limitations (we explain them later on in this paper), they are able to closely mimic the native TM. The use of these TE fabrication methods and the pursuit of fine‐tuning the final scaffold's properties, such as the stiffness or beam morphology, has led to relevant discoveries and drug screening platforms. For instance, hydrogel‐based TM scaffolds have been used to assess the hypothesis that ECM rigidity is a pathophysiological factor in glaucoma (Schlunck et al., 2008). Additionally, hydrogels have also been used to verify that the substratum stiffness alters the mechanotransductors expression (YAP/TAZ route; Dupont et al., 2011) and YAP localization in HTMC, which may modulate the expression of ECM proteins related to glaucoma (Thomasy et al., 2013). SU‐8‐based scaffolds have served as particularly fruitful models to understand AH outflow. They have provided valuable knowledge about the HTMC response under glaucoma‐related drugs. For instance, in 2013, when a SU‐8 scaffold was first fabricated, it was validated by Latrunculin‐B (Torrejon et al., 2013), a drug that disrupts actin filaments reducing transendothelial pressure. The same scaffold served to demonstrate the fibrotic potential of transforming growth factor‐β (TGF‐β2), which can cause elevation in IOP and the ability for a ROCK inhibitor to counteract this effect (Torrejon, Papke, Halman, Bergkvist, et al., 2016; Torrejon, Papke, Halman, Stolwijk, et al., 2016). Finally, it should be mentioned that although TE technological approaches for TM modeling, such as electrospinning (Kim et al., 2009) and 3D bioprinting (Huff et al., 2017), have shown promising results as HTMC hosts, work remains to be done to fully comprehend the behavior and relationship between TM and AH outflow.

## 3 TE APPROACHES TO RECREATING TM

The development of a high‐degree fidelity in vitro scaffolds for the TM presents many challenges. This structure needs to mimic several characteristics to provide an adequate cue for cellular behavior: chemical composition, morphology (Figure 1 shows the layered 3D organization and the porosity gradient ranging from 2 µm, at the inner layers, to 27 µm, at the uveoscleral TM), and mechanical properties. Any mismatch of these variables can result in different cellular behavior. For instance, a more rigid TM may be lead to ocular hypertension (Chang et al., 2017; Last et al., 2011; Schlunck et al., 2008; Thomasy et al., 2013; Wang et al., 2017; described as the most important risk factor for glaucoma development).

There are different technologies available to fabricate scaffolds and fulfill some of the previously described requirements. Thus, the choice of the fabrication method depends on the purpose of the investigation and how desired final structure. In what follows, we present the most relevant fabrication methods, and we point out their pros and cons as well as the objective of the resulting scaffolds.

### 3.1 Photolithography‐based scaffolds

Photolithography is a microfabrication method based on the selective polymerization of photo‐resistant materials (through a photolithographic mask) that have been deposited (homogeneously distributed by spin‐coated) on a flat surface (usually a silicon wafer). This process allows for high resolution and the ability to control pore size and shape enabling the definition of minute features, but only in a 2D or layered fashion. Therefore, this method can be used to either develop a master stamp for the later replica or directly to generate a culture substrate.

Russell et al. used this technique to develop nanopatterned polyurethane substrates to analyze the effect of topographic cues on HTMC. The authors observed that nanopatterned surfaces containing biomimetic length scale features influenced HTMC behavior (Russell et al., 2008). The microtopographic effects were further studied by Zhao et al., who constructed polydimethylsiloxane (PDMS) based microgrooves and micropillars to investigate the influence of these geometries on HTMC monolayers and their hydraulic resistance. In fact, engineered microstructures were found to modify porosity which regulated hydraulic opposition, leading to the hypothesis that an appropriate geometry design will regulate hydraulic resistance (Zhao et al., 2009).

**Figure 3:**
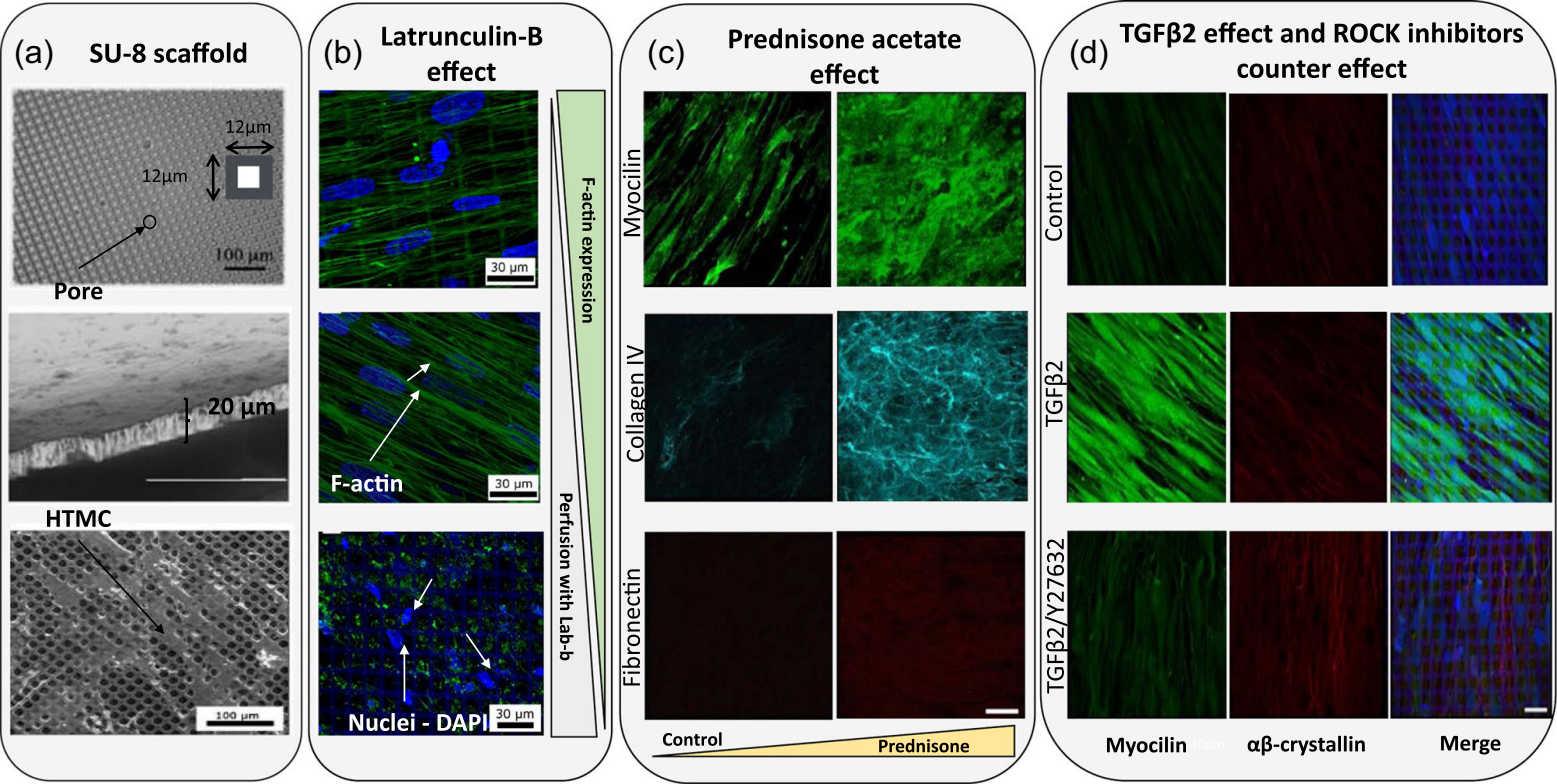
Different studies that use a SU‐8 scaffold as a TM model. (a) Scanning electron microscopy (SEM) images of the microfabricated SU‐8 scaffold. From top to bottom: Pore size of the scaffold, the cross section and SEM micrographs of human TM cells grown in SU‐8 scaffold with a pore size of 12 μm. Reproduced with permission from Torrejon et al. (2013); John Wiley and Sons. (b) Biological response to 2 μM Latrunculin‐B. Confocal images of F‐actin cytoskeleton in green and co‐stained nuclei with DAPI in blue. From top to bottom: before perfusion, after perfusion with medium, and after perfusion with medium and Lat‐B. Reproduced with permission from Torrejon et al. (2013); John Wiley and Sons. (c) Confocal images of myocilin (green), collagen IV (cyan), and fibronectin (red) after perfusion with 300 nM prednisone acetate. Scale bar: 40 μm. Reproduced with permission from Torrejon, Papke, Halman, Bergkvist, et al. (2016), Torrejon, Papke, Halman, Stolwijk, et al. (2016); John Wiley and Sons. (d) Confocal images of human TM protein expression after treatment with 2.5 ng/ml TGFβ2 in the absence or presence of 10 μM Y27632. From left to right, myocilin in green, αβ‐crystallin in red, and merged images. Scale bar = 30 μm. Reprinted from Torrejon, Papke, Halman, Bergkvist, et al. (2016), Torrejon, Papke, Halman, Stolwijk, et al. (2016) (https://www.nature.com/articles/srep38319).

More recently, highly porous, gelatin‐coated SU‐8 scaffolds have been fabricated by means of photolithography (Dautriche, Szymanski, et al., 2015; Torrejon et al., 2012, 2013; Torrejon, Papke, Halman, Bergkvist, et al., 2016; Torrejon, Papke, Halman, Stolwijk, et al., 2016) for drug‐screening applications. These studies were based around the construction of three different pore size micro‐patterned SU‐8 scaffolds made up of arrays of 7, 12, and 15 μm square pores. 12 μm turned out to be the optimum size in terms of supporting HTMC morphology and growth. Figure 3a shows SEM images of these SU‐8 porous membranes, some details of the thickness, and the HTMCs growing on top. We will now look at how this SU‐8 micropatterned scaffold has led to several studies and pharmacological trials. First, the SU‐8 scaffold with HTMC seeded above was validated by perfusing Latrunculin‐B (Lat‐B) through the scaffold. Passing the drug through the SU‐8 structure caused a pressure reduction (Torrejon et al., 2013) because of the Lat‐B depolymerized actin filaments (as shown in Figure 3b). In 2016, these same researchers continued this study to further validate the capacity of this scaffold to steroids response. In Figure 3c, the results of these experiments are shown. Under prednisone acetate presence (a steroid), a fibrotic state was induced, with an overexpression of fibronectin, myocilin, and collagen IV (Torrejon, Papke, Halman, Bergkvist, et al., 2016; Torrejon, Papke, Halman, Stolwijk, et al., 2016). Finally, Figure 3d refers to the study performed to confirm the fibrotic effects of TGF‐β cytokine family, with an overexpression of myocilin and without change in αβ‐crystallin. In this experiment, the antifibrotic properties of a ROCK inhibitor (Y27632) were discovered. This outcome occurred after applying this drug following TGF‐β perfusion, which resulted in lower pressure levels, lower ECM protein accumulation, and αβ‐crystallin overexpression (Torrejon, Papke, Halman, Bergkvist, et al., 2016; Torrejon, Papke, Halman, Stolwijk, et al., 2016).

This 12 μm pore size SU‐8 scaffold was used to identify the SC as the unique vascular endothelium with lymphatic‐like characteristics that functions to mediate IOP outflow homeostasis together with the TM (Dautriche, Szymanski, et al., 2015). This might serve to understand the contribution of the SC to outflow physiology and pathology. A recent study conducted by Tian et. al using this same scaffold has observed that when subjecting human adipose‐derived cells to exogenous vascular endothelial growth factor C (VEGF‐C), shear stress and coculturing them with HTMC can provide mechanical and cellular cues necessary for human Schlemm's Canal cells (HSC) differentiation. Thus, overcoming the isolation difficulties of primary HSC, is critical for outflow physiology and glaucoma understanding (Tian et al., 2020).

Overall, the dimensions of the SU‐8 platform resembled a 2D version of the native TM. Also, the cultured HTMC exhibited correct physiological activity, that is, it responded to drugs. Whilst the pore size and the beam diameter resemble the TM tissue, the scaffold design does not provide the desired 3D structure. This assembly is similar to commercially existing membranes (Dai & Li, 1999; Pedrigi et al., 2011; Perkins et al., 1988; Underwoord et al., 1999) with uniform and well‐controlled patterns. Moreover, in a separate study where SU‐8 was used, scaffolds with a similar thickness of 25 µm were achieved yielding an elastic modulus of 2.2 ± 0.1 GPa (Gao et al., 2010). These results are considerably stiffer than the human TM, which hinders the applicability of SU‐8 as a TM model since the unfavorable effects of a highly stiff environment on HTMC are well known (Chang et al., 2017; Last et al., 2011; Schlunck et al., 2008; Thomasy et al., 2013; Wang et al., 2017). Moreover, photolithography‐based scaffolds require a clean room, which may not always be available, and typically need to be coated or chemically modified with ECM components found at the JCT‐SC border (Dautriche, Tian, et al., 2015).

### 3.2 Electrospun nanofiber‐based scaffolds

Electrospinning is a fabrication technique based on the electrostatic interactions (repulsion forces) between a polymeric solution and the extrusion needle to generate micro/nano‐polymer fibers (Huang et al., 2003; Szentivanyi et al., 2011). Its primary advantages are its ability to generate highly porous structures mimicking the fibrous components of the native ECM, the fact that it yields large production batches, and ensuring high degrees of repeatability (Feltz et al., 2017; Matthews et al., 2002; Norman & Desai, 2006). This technique has been successfully employed for the generation of polymeric scaffolds to recreate bone (Prabhakaran et al., 2009), skin (Kumbar et al., 2008; H. Lu et al., 2012), cardiac (Muniyandi et al., 2020), or even vascular tissues (Kai et al., 2015).

Moreover, the electrospinning technique enables the generation of fiber mats with varying diameter and pore sizes by changing the operation parameters, such as voltage, needle‐collector distance, type of materials, and their concentration. Among possible materials for ophthalmology scaffolding, the most used synthetic polymers are polyvinyl pyrrolidone, polyvinyl alcohol, polyglycolide acid, polylactic acid, poly(lactide‐*co*‐glycolide), polyethylene oxide, polypropylene oxide, and polycaprolactone (PCL; Calles et al., 2015; Crouch et al., 2021).

**Figure 4:**
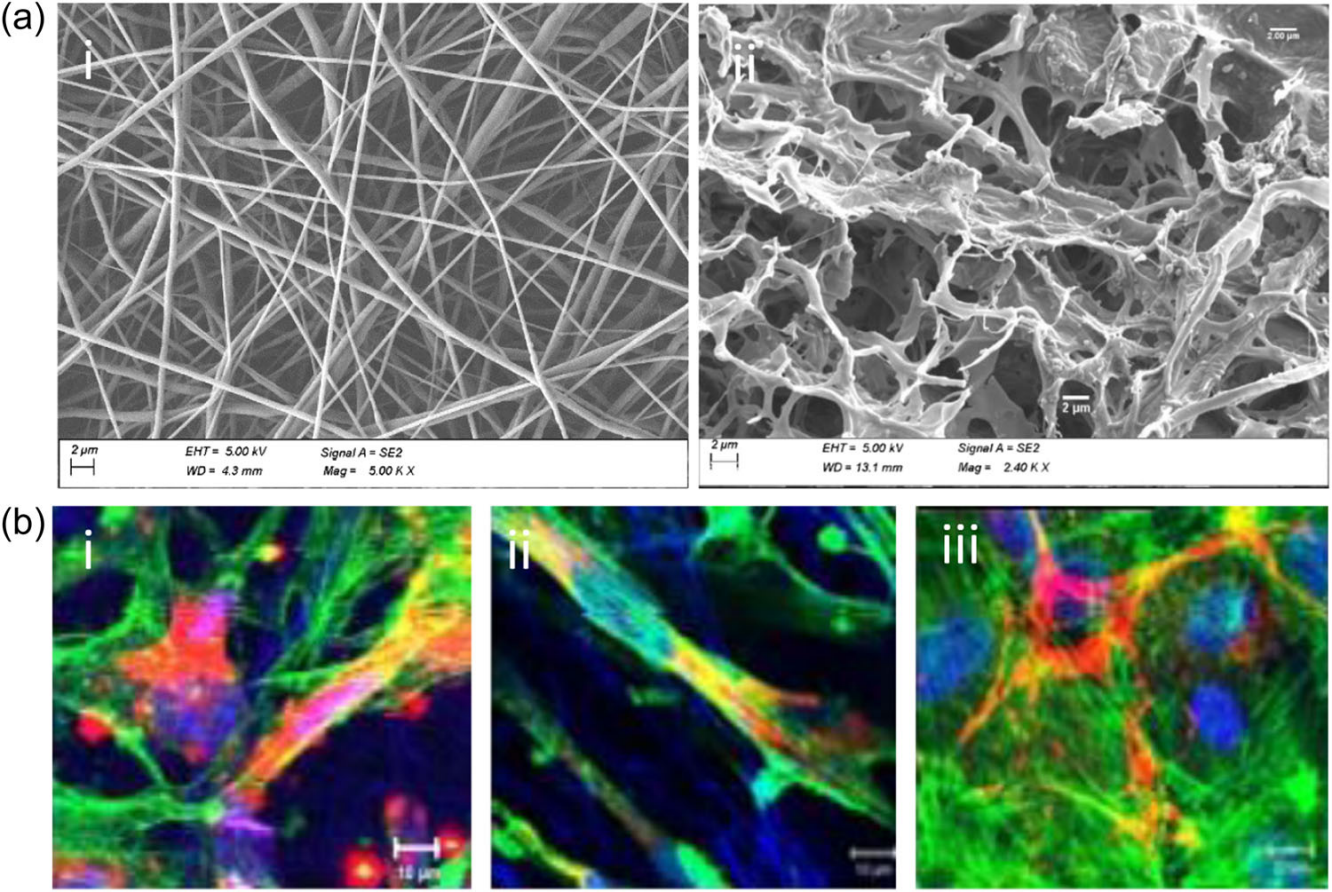
Electrospinning‐based scaffolds for TM tissue. (a) SEM images showing similarities between: (i) PCL electrospinning‐based scaffold and (ii) native human TM tissue. (Scale bar: 2 µm) (B) Laser scanning confocal microscopy images of TM cells over nanofibers (i) non‐aligned PCL, (ii) aligned PEUU, and (iii) glass, respectively. Nuclei of TM cells in blue (DRAQ 5TM), actin filaments in green (Alexa Fluor 488 phalloidin®), and the ECM materials in red (Alexa fluor 555®). PEUU, poly‐etherurethane urea; SEM, scanning electron microscopy; TM, trabecular meshwork. Reprinted from Kim et al. (2009). Thirteenth International Conference on Miniaturized Systems for Chemistry and Life Sciences.

Despite the use of nanofiber mats for ocular research platforms (Da Silva et al., 2015; Kador et al., 2013; Omer & Zelkó, 2021), there is little literature focused on TM‐related investigations. A study performed by Kim et al., reports the fabrication of 3D human TM using electrospun micro/nanofibers. Cell morphology on electrospun polymer nanofibers was similar to the native environment (Kim et al., 2009, 2011). It is clear from these results that PCL electrospun nanofibers resemble the native TM tissue (Figure 4a). In the aforementioned work of Kim et al., random PCL and aligned poly‐etherurethane urea fibers were built and HTMC was cultured for 10 days to study cell proliferation (Figure 4b; Kim et al., 2009). This study provided an in vitro model to study the AH outflow facility and the role of TM tissue, and presented several advantages over the currently used two‐dimensional TM models. With the same aim, another group confirmed PCL scaffold's appropriateness for HTMC proliferation and dexamethasone response after a thorough mechanical characterization (Izagirre et al., 2020). In this study, a scaffold holder together with a pressure‐controlled system was designed, ultimately showing promising results to carry out drug testing experiments.

All in all, electrospun fibers have proven to be a suitable medium to host HTMC and have enabled the correct cellular behavior. Nanofiber structures are ideal for cell attachment (Kwon & Kidoaki, 2003), drug loading (Sang et al., 2009), or protein absorption (Ganesh & Ingavle, 2014). The easy tuning of fabrication parameters offers a wide range of final morphologies, which is known to modulate cellular response. For instance, small pores could impede cellular infiltration (Khorshidi et al., 2014), which can compromise the scaffold's fate. In general, electrospun scaffolds, as the previously mentioned photolithography‐based structures, will present high elastic modulus (around GPa; Kennedy et al., 2017) and will need a surface treatment (coating or plasma oxygen) to enhance cellular attachment. However, these micro/nano‐based constructs provide directionality and a 3D environment for HTMC, which will properly evoke the native TM.

### 3.3 Hydrogel‐based scaffolds

The application of hydrogels in scaffold fabrication is a thorough and well‐documented topic and makes up a particularly important niche in the TE field. This comes from the design advantages that hydrogels can provide as they can yield great three‐dimensional (3D) environments with different stiffness levels and morphologies, biocompatibility, and structural maintenance integrity (Chai et al., 2017; Dhandayuthapani et al., 2011; Geckil et al., 2010; Leijten et al., 2017).

Hydrogels can be created from a variety of synthetic or natural materials, with approaches based on natural polymers being the most widely adopted in the literature of TE (Lee & Mooney, 2001). Considering the ECM of the TM; collagen, fibrin, and elastin have been employed as attachment factors for HTMC to study specific functions and interactions (Liton et al., 2014; Wudunn, 2009; Zhou & Zhang, 1996). Whaduthanthri et al. developed a hydrogel peptide called MAX8 to bioengineer an in vitro 3D TM scaffold, which could potentially be injected as tissue scaffold in glaucoma patients after trabeculectomy. The initial viscosity of the gel‐cell construct was 35 ± 4 Pa, which increased significantly as a result of cell growth (1374 ± 22 Pa). These results did not alter the shear‐thinning property of the scaffold and indicated a good environment for HTMC growth, being potentially employable as an injectable implant (Waduthanthri et al., 2019).

**Figure 5:**
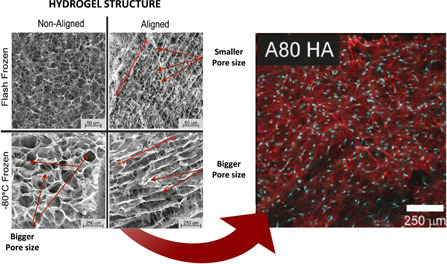
Pore size effects. Larger pores indicate higher proliferation levels and fibronectin expression. Left: SEM images of scaffolds frozen in non‐aligned and aligned configurations. Red lines indicate the direction of the pore alignment. Scale bar: 50 μm for the flash frozen and 250 μm for the −80°C frozen scaffolds. Right: Confocal micrographs of hyaluronic acid scaffolds after 2 weeks of culture. Fibronectin (red) and nuclei (blue). Reproduced with permission from M. J. Osmond et al. (2020); John Wiley and Sons.

**Figure 6:**
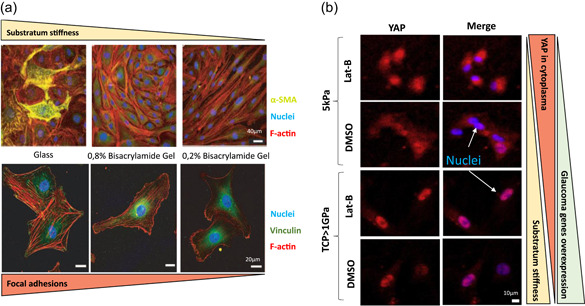
Substratum stiffness effects. (a) Substrate rigidity modulates α‐SMA localization. HTM cells grown on collagen‐coated coverslips and collagen‐coated stiff (0.8% bis‐acrylamide) or (0.2% bis‐acrylamide) polyacrylamide gels for 10 days. Composite images depict F‐actin in red, α‐SMA in green, and cell nuclei in blue. Some cells exhibit α‐SMA positive stress fibers on stiff polyacrylamide gels, whereas intense staining is observed on glass coverslips. Scale bar: 40 μm. Bottommost images indicate higher focal adhesions on stiffer substrates. F‐actin (red), vinculin (green), and nucleus (blue). Scale bar: 20 μm. Reproduced with permission from Schlunck et al. (2008) Association for Research in Vision & Ophthalmology (ARVO). (B) Substratum stiffness and Lab‐B alters nuclear/cytoplasmic localization in HTM cells. HTMC stained for YAP (red) and counterstained with DAPI (blue). YAP localization in HTMC is mixed between nuclear and cytoplasmic. Nuclear localization is more pronounced on TCP (>1 GPa) than on the 5 kPa hydrogel. With Lat‐B treatment, nuclear YAP localization is decreased on the 5 kPa hydrogels but increased on TCP, which induces higher probabilities to overexpress glaucomatous genes. Scale bar: 10 μm. Reproduced with permission from Thomasy et al. (2013). Elsevier.

The versatility hydrogel molding offers, allows pore size alteration. Freeze‐casting methods have been used to build a 3D collagen and collagen‐chondroitin sulfate (CS) scaffolds (Kelley, 2008). In this study, pore sizes were 10.25 ± 5.1 µm for collagen scaffolds, 9.48 ± 4.7 µm for the collagen‐CS and elastic moduli 6.71 ± 3.2 kPa and 6.73 ± 1.7 kPa, respectively. These biocompatible materials allowed the correct growth of porcine TM cells (M. Osmond et al., 2017). Recently, the same authors reported that bigger pore sizes and their alignment, enhanced the TM cellular activity as much as the introduction of glycosaminoglycans (Figure 5; M. J. Osmond et al., 2020). The mechanical properties of hydrogels are also important, since the adhesion and gene expression of cells are strongly related to the environment's stiffness (Lee & Mooney, 2001). Schlunk et al. prepared polyacrylamide substrates with different rigidities and collagen‐coated tissue culture plastic. Their goal was to study the effects these different rigidities had on HTMC cells. Figure 6A shows how an increase in substrate stiffness enhances focal adhesions, fibronectin, and α‐SMA expressions. This was enough to assess substrate rigidity as an HTMC behavior modulator and as a possible pathophysiologic factor in glaucoma (Schlunck et al., 2008). In another study, Thomasy et al. fabricated a polyacrylamide hydrogel that successfully re‐enacted the stiffness of normal (4–5 kPa) and glaucomatous (75 kPa) human TM. They compared it to tissue culture polystyrene (1 GPa) and seed primary HTMC. This study was able to prove that substratum stiffness alters YAP/TAZ expression placing its expression in the nucleus on rigid surfaces. In fact, Lat‐B treatment was shown to increase YAP expression inside the nucleus of the aforementioned hard solid layers, which resulted in a favorable environment for glaucoma to arise (Figure 6b; Thomasy et al., 2013).

The latest research on the use of hydrogels as scaffolds revolves around mixing normal donor‐derived HTMC with collagen type I, elastin‐like peptide, and hyaluronic acid. The purpose of the study was to test the photo‐crosslinked polymeric hydrogel as a suitable human TM model. Indeed, the scaffold responded with glaucomatous conditions (hydrogel stiffening) when dexamethasone was applied and reacted to ROCK inhibitor with therapeutic effects (Li et al., 2021). This hydrogel is tuneable in stiffness, a valuable characteristic to study the roles of YAP/TAZ in HTMC in response to a stiffened matrix and TGFβ2 (Li et al., 2022). They have confirmed that an increase in stiffness elevates YAP/TAZ nuclear localization, which may exacerbate disease pathology conditions. In a similar manner, Bouchemi et al. designed a new culture model of primary HTMC using Matrigel® to emulate the structure of in vitro 3D‐TM, with the goal of investigating the pro‐inflammatory effect of benzalkonium chloride in 3D human TM cultures (Bouchemi et al., 2017).

Overall, compared to photolithography and electrospinning, hydrogel molding offers higher control over the morphology (pore sizes), stiffness (similar to the native TM), and the 3D environment, while also maintaining the structural integrity. In addition, the materials used in hydrogel molding, like elastin, collagen, or fibrinogen, offer higher cellular integrity as they belong to the ECM of the native TM. In general aspects, hydrogels are perfect for stiffness and environmental‐change studies, indicating prosperous advances in terms of HTMC reactions over these environment alterations. However, they are difficult to handle and, unlike photolithography‐based scaffolds, their use in pressure and drug testing studies is limited (Tirendi et al., 2020; Waduthanthri et al., 2019).

### 3.4 3D bioprinted scaffolds

3D bioprinting is experiencing a significant growth in popularity due to the manufacturing speed and precise geometry with which materials can be printed (Matai et al., 2020). To date, a variety of bioprinting strategies have been proposed to engineer different models of interest, including those based on stereolithography, extrusion, and droplets (Heinrich et al., 2019). 3D bioprinting can produce a great variety of architectural patterns on a wider array of biomaterials. However, this technique's resolution is not yet high enough to accurately reproduce the complex porous structure of the in vivo TM nor its thickness. In consequence, 3D bioprinting has not yet established itself as a fabrication method for TM models. Nonetheless, this method has been used in some research studies in which sodium alginate and methacrylate gelatin were used to optimize the pore resolution and the printing parameters. Although they were able to print the hydrogel scaffolds and seed them with living cells, they concluded that more work is necessary to determine the optimum printing parameters (Huff et al., 2017). Compared with extrusion‐based 3D printing, stereolithographic 3D bioprinting may be a more promising method for building future TM in vitro models because of its current higher resolution and the absence of mechanical extrusion (R. Lu et al., 2020).

As a whole, 3D bioprinting is still on the come‐up and despite its promise and application in the TE field, there is still a long way to go in 3D human TM modeling.

## 4 ENGINEERING OUTFLOW PHYSIOLOGY PLATFORMS

Engineered outflow platforms seek to emulate the AH outflow physiology and replicate the responses seen in clinical cases. General perfusion studies of outflow physiology can provide information about the hydraulic conductivity keeping a constant pressure and measuring the flow rate. Similarly, outflow facility can also be determined by keeping the flow rate constant to measure the pressure, or sequentially increasing the flow rate to record the pressure variations at each speed. Table 2 summarizes a set of studies (mentioned below), focusing on the hardware of the designs, human TM scaffolds, working parameters, and the administrated drugs.

**Table 2:** Characteristics of the perfusion studies

Perfusion system	Scaffold and fabrication method	Flow (µl/min)	No. of cells—culture time before exposition	Treatment	Results	Refs.
		time (h)				
		pressure (mmHg)				
Millicell housing support Calibrated flow meter monitor	HATF filters 50–55 µm thick 13 mm diameter	5 mmHg pressure differential Measured Hydraulic Conductivity (HC): Lp = Q/P*A	HTMC 10^4^ cells/ml in HATF filter for 21 days	1 h perfusion with Chitosan B	HC HATF alone: 50.5 µl/min/mmHg/cm^2^ HATF cell monolayer: 1.1 µl/min/mmHg/cm^2^	Perkins et al. (1988)
	Nylon Filters Milipore 2.5 cm diameter	5 mmHg pressure differential Measured Hydraulic Conductivity (HC): Lp = Q/P*A 1 h perfusion or 5 days	HTMC 10^6^ cells/ml for 10 days over filters	No cells No treatment No treatment (DMEM) Epinephrine 10^−5 ^mol/L in DMEM Dexamethasone 10^−6 ^mol/L in DMEM	HC: 32–34 µl/min/mmHg/cm^2^ HC: 9–11 µl/min/mmHg/cm^2^ HC: 12–14 11 µl/min/mmHg/cm^2^ HC (1 h): 21–22 µl/min/mmHg/cm^2^ HC (5 days): 2–3 µl/min/mmHg/cm^2^	Dai and Li (1999)
Flow column Flowmeter Pressure gauges Resistor with a resistance of 0.5 mmHg/µl/min	Permeable methylcellulose filters 0.45 mm pores and area of 0.6 cm^2^	Fluid column: 5.5 mmHg HC = Q/P sensor/Area	HTMC and HSC cells 5 × 10^4^ cells/cm^2^ up to 7 weeks	No treatment − 2 weeks culture − 7 weeks culture Dexamethasone 500 nM − 7 weeks of treatment	Hydraulic conductivity of: Filters alones: 0.04 mmHg/µl/min/cm^2^ HTMC: 1.0 mmHg/µl/min/cm^2^ HSC: 0.5 mmHg/µl/min/cm^2^ HTMC: 3.0 mmHg/µl/min/cm^2^ HSC: 1.5 mmHg/µl/min/cm^2^ HTMC: 0.5 mmHg/µl/min/cm^2^ HSC: 0.6 mmHg/µl/min/cm^2^	Underwood et al. (1999)
Computer controlled syringe pump Pressure transducer Membrane insert adapter Microscope	Transwell permeable polyester filter membranes 0.4 µm pore 12 mm diameter 4 × 10^6^ pores/cm^2^	200 µl/min 25 min perfusion 2–6 mmHg	Human Schlemm Canal (HSC) Endothelial Cells 4.5 × 10^4^ cells/cm^2^ for 2 days		With higher pressures bigger became the vacuoles Hydraulic conductivity: 1.08–2.98 µl/min/mmHg/cm^2^	Ryan et al. (2010)
Flow pressure measurer Perfusion chamber Scaffold holder Pressure transducer (Edwards Lifesciences)	SU‐8 photolithography Thickness 20 µm Pore: 12 µm Beam width: 7.3 µm	Flow rates: 2, 10, and 40 µl/min	HTMC 4 × 10^4^ cells/cm^2^ for 14 days	Flow of 40 µl/min for 4 h with: − No treatment − Treatment with 2 µM Latrunculin‐B	Outflow facility: 4.7 µl/min/mmHg	Torrejon et al. (2013)
		24 h perfusion			Transmembrane pressure:	
		Transmembrane pressure:			Before Lat‐B: 9 mmHg	
		− With cells: 8 mmHg − No cells: 0.3 mmHg			After Lat‐B: 0.6 mmHg	
		Flow rates: 2, 4, 8, 10, 14, and 20 µl/min 6 h perfusion	HSC cells 5 × 10^4^ cells/cm^2^	2.5 ng/ml TGF‐β2 − No treatment − With treatment	HSC Outflow facility: 0.046 µl/min/mmHg/mm^2^ HTMC Outflow facility: 0.104 µl/min/mmHg/mm^2^ No data available	Dautriche, Tian, et al. (2015)
		Flow rates: 2, 10, 20, and 40 µl/min 6 h perfusion	HTMC 4 × 10^4^ cells/cm^2^ for 14 days	2.5 ng/ml TGF‐β2 10 µM ROCK inhibitor Y27632 TGF‐β2 (2.5 ng/ml) + Y27632 (10 µM)	Increased actin stress‐fiber and ECM proteins expression Increased transcellular pressure Outflow facility: 0.049 µl/min/mmHg/mm^2^ Shorter collagen fibers aligned fibronectin fibers Outflow facility: 0.23 µl/min/mmHg/mm^2^ Reduction of myocilin, increase of αB‐crystallin expressions Outflow facility: 0.17 µl/min/mmHg/mm^2^	Torrejon, Papke, Halman, Bergkvist, et al. (2016), Torrejon, Papke, Halman, Stolwijk, et al. (2016)
		2, 10, and 40 µl/min 6 h perfusion	HTMC 4 × 10^4^ cells/cm^2^ for 14 days	300 nM Prednisone acetate − No treatment − With treatment	Outflow facility: 0.131 µl/min/mmHg/mm^2^ Outflow facility: 0.093 µl/min/mmHg/mm^2^ Increased myocilin expression and ECM material Increase crosslinking	Torrejon, Papke, Halman, Bergkvist, et al. (2016), Torrejon, Papke, Halman, Stolwijk, et al. (2016)
		2, 4, 8, and 16 ml/min 5 h perfusion	5 × 10^4^ cells/scaffold for 11 days − Co‐culture of Adipose tissue‐derived stem cells (ADSC), HSC and HTMC − HTMC/HSC − HTMC	With Dexamethasone 100 nM − HTMC/HSC − HTMC/ADSC/HSC No treatment − HTMC − HTMC/HSC − HTMC/ADSC/HSC	Outflow facility:	Tian et al. (2020)
					− Day 3: 0.11 µl/min/mmHg/mm^2^ − Day 7: 0.08 µl/min/mmHg/mm^2^	
					Outflow facility:	
					− Day 3: 0.14 µl/min/mmHg/mm^2^ − Day 7: 0.08 µl/min/mmHg/mm^2^	
					Outflow facility: 0.14 µl/min/mmHg/mm^2^ 0.09 µl/min/mmHg/mm^2^ 0.07 µl/min/mmHg/mm^2^	
Syringe pump Swinnex Filter holder Nalgene® tubing BLPR2 differential pressure transducer	Hydrogel Max 8B with no cells. G′ = 335 Pa and G″ = 28 Pa With cells after 7 days of growth. G′ = 1374 Pa and G″ = 74 Pa	Constant 3 µl/min 48 h perfusion	HTMC 2 × 10^5^ cell/ml for 7 days	100 nM Dexamethasone 3 µl/min for 72–96 h	Internal pressure in the perfusion chamber augmented 85% after dexamethasone	Waduthanthri et al. (2019)
Close circuit Single flow reactor Live Box 1 IVTechs.r.l Culture chambers connected to a peristaltic pump	Corning Matrigel	70 µl/min 72 h and 168 h perfusion	HTMC	500 µM of H_2_O_2_ for 2 h and 22 h of recovery	Study of oxidative stress on human TM where a pro‐inflammatory response was observed	Tirendi et al. (2020)

Outflow studies began around 1988, when HTMCs were first cultured on filters. This study introduced the very first perfusion holder together with a pressure/flow circuit at a constant pressure differential to measure the hydraulic conductivity of cultured monolayers of HTMC and to evaluate the effects of cytochalasin (CB) on the hydraulic conductivity (Perkins et al., 1988). These outcomes factored into a later publication, where Transwell® permeable filter membranes were used as a TM scaffold with 0.4 µm pore diameter and perfused in the basal‐to‐apical direction. The goal of this last study was to perform time‐lapse or perfusion fixation studies between pressure drops from 2 to 6 mmHg for 25 min and to analyze the biomechanics of giant vacuole formation in cultures of human SC cells (Ryan et al., 2010). The perfusion system developed by the group of Yubing Xie has also been used in several other publications. Their SU‐8 scaffold enabled the continuous pressure monitoring at a serial of flow rates, therefore, allowing to determine the simulated outflow facility, and the identification of biophysical changes while different drugs (Lat‐B, ROCK inhibitors, TGFβ2) were perfused (Dautriche, Szymanski, et al., 2015; Torrejon et al., 2012, 2013; Torrejon, Papke, Halman, Bergkvist, et al., 2016; Torrejon, Papke, Halman, Stolwijk, et al., 2016).

Recently, the previously mentioned MAX8 peptide‐hydrogel scaffold was tested in a perfusion chamber with a differential pressure transducer for constant monitoring. The scaffold holder of 13 mm in diameter (Swinnex filter holder, Sigma‐Aldrich) successfully hosted the experiment, which was designed to showcase the usability of this hydrogel‐peptide as a 3D TM scaffold (Waduthanthri et al., 2019). The latest work on this topic used a single flow close‐circuit bioreactor, with a 3D Matrigel® as an artificial TM scaffold. The hydrogel was held on a chamber previously designed by these authors (Giusti et al., 2014) and the whole setup preserved the physiological conditions of the HTMC and allowed the analysis of the role of oxidative stress in promoting the degeneration of TM tissue (Tirendi et al., 2020). Unfortunately, despite the ability of hydrogels to mimic the mechanical properties of TM ECM, conducting perfusion studies can be difficult with hydrogels. In the studies mentioned above, HTMC were cultured over hydrogels to measure pressure changes at constant flow rates (hydraulic conductivity), but perfusion studies at different flow rates to measure the outflow facility remains a challenge.

In general terms, the previously mentioned studies share similar hardware components, including syringe pump, pressure transducers (with operating ranges from −50 to 300 mmHg and sensitivity of 5 μV/V/mmHg), a computer for system controlling and data acquisition, and a scaffold holder for HTMC seeding. Most of them follow the same working principle, which consists in applying small flow rates (2–70 μl/min) in the apical‐to‐basal direction. These designs can measure alterations in the transmembrane conductivity, caused by modifications in HTMCs' behavior when they are exposed to different drugs. Both pressure ranges and flow rates fulfill the physiological requirements of this particular tissue.

Despite the good performances of these systems, they present some drawbacks such as the limited duration of the study (see Table 2) due to hypoxia, media change or sterility, the number of samples per experiment, and the size of the scaffold holder. The scaffold size compromises the amount of genetic and protein material available for the subsequent analysis. These facts are necessary to confirm the effects of pressure, drugs, or shear stress on cellular activity. Overall, given that most of these designs are ad hoc, there are many different and hard to account for variables that may cause differences in experimental results. Standardization of a bioreactor system would provide the field homogeneity on experimental performance.

## 5 CONCLUSIONS

In reality, glaucoma pathophysiology represents the failure of multiple ocular systems. Naturally, only considering the dysfunctions of a single tissue will leave aside important aspects of this illness. In this context, the study of the TM and its drainage capacity seems to be a cornerstone in glaucoma development. Thus, in this review, we have compared available scaffold fabrication methods for human TM modeling on one side, and we have surveyed the AH outflow platforms on the other. A chronological overview of technological advances of these models is provided, emphasizing those which have led to important milestones to glaucoma research.

Different scaffolds and technologies have been used to recreate the TM, including traditional polymeric filters, SU‐8 membranes, electrospun nanofibers, and hydrogels. Researchers have been able to accurately control the morphological characteristics (porosity and beam thickness) by providing 2D and 3D environments. Regarding mechanical characteristics, only hydrogels have allowed direct control of the stiffness of the scaffold. Aside from these techniques, the rapid evolution of 3D bioprinting may also lead to the creation of a model in which the appropriate TM scaffold requirements are fully satisfied.

There is a limited number of AH outflow platforms in the literature, and most of them share the same hardware design and working principle, which yield appropriate conditions for the cells to react to drugs as intended. However, important challenges must first be resolved if the functionality of these bioreactors is to be improved. Examples of such challenges are enabling long‐term assays to allow slow biochemical processes to affect the HTMC performance or providing high throughput systems that allow multiple parallel drug screening. Even more, a standardized platform should be agreed on to unify the results in this field. To sum up, technological advances have enabled significant progress in TM research and its relationship to glaucoma, and they will, without a doubt, continue promoting breakthroughs.

## AUTHOR CONTRIBUTIONS

All authors agreed on the order in which the names are listed in the manuscript.

## CONFLICT OF INTEREST

The authors declare no conflicts of interest.

## Data Availability

Data sharing is not applicable to this article as no new data were created or analyzed in this study.
